# Capillary Electrophoresis as a Complementary Analytical Tool for the Separation and Detection of Nanoplastic Particles

**DOI:** 10.1021/acs.analchem.4c00822

**Published:** 2024-04-30

**Authors:** Carlos Adelantado, Blanca H. Lapizco-Encinas, Jan Jordens, Stefan Voorspoels, Milica Velimirovic, Kristof Tirez

**Affiliations:** † Flemish Institute for Technological Research (VITO), Boeretang 200, 2400 Mol, Belgium; ‡ Microscale Bioseparations Laboratory and Biomedical Engineering Department, Rochester Institute of Technology, 160 Lomb Memorial Drive, Rochester, New York 14623, United States

## Abstract

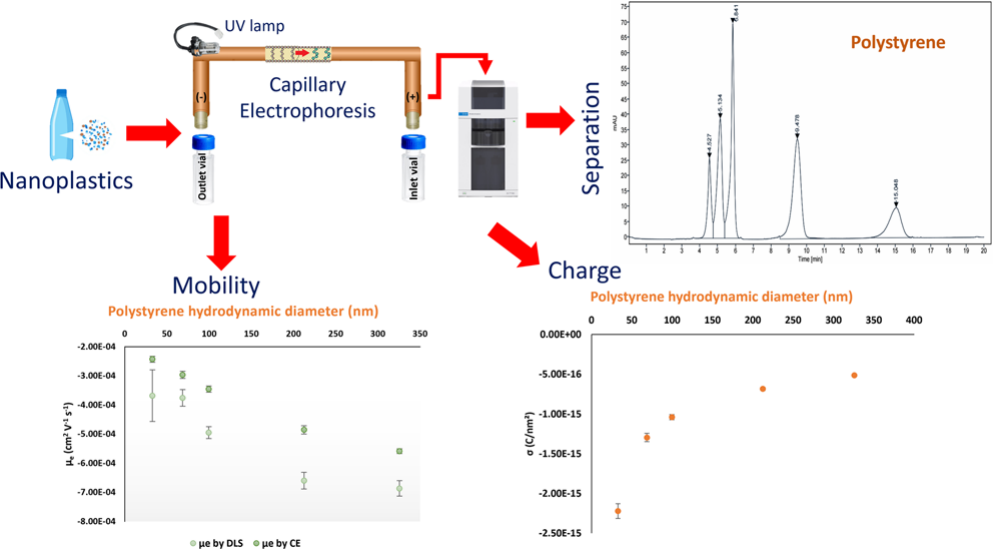

Capillary electrophoresis (CE) is presented as a technique for the separation of polystyrene nanoparticles (NPs, particle diameters ranging from 30 to 300 nm) through a bare fused silica capillary and ultraviolet detection. The proposed strategy was also assessed for other types of nanoplastics, finding that stronger alkaline conditions, with an ammonium hydroxide buffer (7.5%, pH = 11.9), enabled the separation of poly(methyl methacrylate), polypropylene, and polyethylene NP for the first time by means of CE for particle diameters below 200 nm. Particle behavior has been investigated in terms of its effective electrophoretic mobility, showing an increasing absolute value of effective electrophoretic mobility from the smaller to the larger sizes. On the other hand, the absolute value of surface charge density decreased with increasing size of NPs. It was demonstrated and quantified that the separation mechanism was a combination of linear and nonlinear electrophoretic effects. This work is the first report on the quantification of nonlinear electrophoretic effects on nanoplastic particles in a CE system.

## Introduction

Nanoplastics (1 nm – 1 μm) are increasingly present in the environment and daily life products, and fast, reliable, and sensitive approaches are required for an accurate analysis across diverse environmental matrices. As capillary electrophoresis (CE) has emerged over the last decades as a suitable technique for nanomaterial identification and quantification,^1^ it can also be considered as a method of choice for the analysis of nanoplastics. It is likely to establish a critical comparison of several aspects between CE and chromatographic modalities, namely, high-performance liquid chromatography (HPLC), capillary liquid chromatography (CLC), size-exclusion chromatography (SEC), hydrodynamic chromatography (HDC), or diffusion-based techniques such as asymmetrical flow field-flow fractionation (AF^4^). The use of electroosmotic flow (EOF), which features a flat flow, provides for nondispersive liquid transport, in contrast with the pump-driven parabolic flow profile in HPLC systems; the former leads to narrow peaks and better resolution, and the number of theoretical plates in CE is greater. Additionally, CE-based approaches can be carried out making use of thin capillaries as separation compartments. When compared with CLC, SEC, and HDC, it can be inferred that overall process time is shorter and solvent consumption is lower (tens–hundreds of μL), placing CE in an advantageous position. The main difference of CE, distinguishable from the aforementioned techniques, is the existence of a whole charge as a carrier force for NPs through the capillary; this goal is typically achieved by provoking interactions between the modified particle surface and the background electrolyte.^2^

The usefulness of CE in nanomaterials for characterization, screening, direct analysis of actual samples, and hyphenation with state-of-the-art detection devices has decisively contributed to the progress of CE in analytical nanometrology. More importantly, as approaches to be considered in the field of analysis of nanoplastics, there have been a few studies on the CE potential to separate polystyrene (PS) NPs of various diameters^3−6^ with different detection devices. CE systems have been conventionally analyzed in terms of linear electrophoresis, which is the electrophoretic migration of particles in the weak field regime, where the electrophoretic mobility is independent of the electric field magnitude. The fundamental understanding of linear electrophoresis was established during the 20th century where many electrophoresis-based techniques were established.^7^ Linear electrophoresis is an efficient method for discriminating particles by their electrical charge, and it has been successfully employed in numerous studies for the separation of macromolecules. However, linear electrophoresis may not be able to discriminate analytes close in size or shape.^8^

Nonlinear electrophoresis effects occur under conditions of strong electric fields and particles of sufficient size, which is the case for some of the NPs herein studied. The first reports on nonlinear electrophoresis (EP_NL_) were published by Dukhin in the 1970s;^7,9^ the majority of these reports were theoretical studies without experimental demonstrations, which perhaps hindered the widespread use of nonlinear electrophoresis. In 2011, it was further confirmed by Mishchuk and Barinova that experimental data on nonlinear electrophoresis were scarce.^10^ The past decade has witnessed an important resurge in the study of nonlinear electrophoresis,^7^ and several recent reports have demonstrated the significant effects of nonlinear electrophoresis (EP_NL_) on particle electromigration.^11−17^ These reports proved that particles could experience two distinct electrophoretic forces, a linear force and a nonlinear force, depending on the system conditions, including the strength of the electric field. Furthermore, EP_NL_ is able to discriminate particles by size and shape, which is not possible with only linear electrophoresis effects.^8^ The effects of the EP_NL_ force become significant at higher local electric fields, and these effects are in the same direction as the linear electrophoresis effects. Thus, in this study, the effects of EP_NL_ are an additional electrophoretic force pushing the particles toward the capillary inlet, increasing particle retention time in the capillary.

In view of the proposed background, it has been hypothesized that CE coupled with an appropriate detection device may be a fit-for-purpose analytical technique to identify and quantify nanoplastics.^18,19^ This technique is called for providing deeper insights into size distribution and surface charge functionality of the aforementioned particles in the matrix subject to study. Therefore, CE in combination with a ultraviolet–visible (UV–vis) spectrophotometric diode-array detector (DAD) has been evaluated for the nanometrological approach herein described in which separation of various types of nanoplastic particles with different diameters has been dealt with, together with a study of electrophoretic parameters calculated from both experimental measurements on CE and laser Doppler velocimetry (LDV) as a supplementary technique. Furthermore, the present study also represents the first report where the effect of nonlinear electrophoretic migration in a CE system is quantified and analyzed, offering an extra effect to be exploited in separation processes.

## Experimental Section

### Nanoparticles

Nonfunctionalized PS spheres of different particle diameters (30, 60, 90, 200, 300 nm) were purchased from Distrilab (The Netherlands), as 1% m/v solid. Nonfunctionalized polyethylene (PE) (65 nm), polypropylene (PP) (54 nm), and poly(methyl methacrylate) (PMMA) (50, 100, 200 nm) spheres, also as 1% m/v solid, were supplied by Lab 261 (Palo Alto, CA). Information on the preparation of buffer solutions is included in the Supporting Material. All particles used in this study are listed in Table 1.

**Table 1:** Particle Features According to Specifications and Experimental Measurements According to Optimized Experimental Conditions throughout This Research

particle	diameter by the supplier in nm	hydrodynamic diameter ± SD (*n* = 5) in water (DLS) in nm	hydrodynamic diameter ± SD (*n* = 5) in buffer (DLS) in nm	ζ-potential (mV)	linear electrophoretic mobility × 10^–4^ (cm^2^ V^–1^ s^–1^)	nonlinear electrophoretic mobility × 10^–10^ (cm^4^ V^–3^ s^–1^)
**PS**	31 ± 3, *k* = 2^a^	32.8 ± 0.2	35.2 ± 0.9^c^	–44.3 ± 0.6^c^	–3.08 ± 0.04^c^	–2.79 ± 0.17^e^
**PS**	62 ± 3, *k* = 2^a^	68.6 ± 0.9	69.9 ± 1.6^c^	–50.9 ± 0.9^c^	–3.53 ± 0.06^c^	–2.32 ± 0.09^e^
**PS**	92 ± 3, *k* = 2^a^	99.6 ± 0.5	95.8 ± 0.5^c^	–57.9 ± 2.4^c^	–4.02 ± 0.17^c^	–2.42 ± 0.21^e^
**PS**	202 ± 4, *k* = 2^a^	212.4 ± 0.9	207.8 ± 1.7^c^	–71.9 ± 4.9^c^	–4.99 ± 0.25^c^	–1.39 ± 0.21^e^
**PS**	303 ± 6, *k* = 2^a^	325.6 ± 3.2	305.5 ± 12.6^c^	–86.3 ± 9.5^c^	–5.99 ± 0.10^c^	g
PMMA	38^b^	37.3 ± 0.7	h	–36.0 ± 3.3^d^	–2.50 ± 0.23^d^	–4.73 ± 0.46^f^
PMMA	103^b^	125.2 ± 0.7	131.0 ± 0.7^d^	–51.4 ± 2.3^d^	–3.57 ± 0.16^d^	–6.47 ± 0.33^f^
PMMA	219^b^	207.3 ± 0.5	217.9 ± 2.2^d^	–62.2 ± 2.8^d^	–4.32 ± 0.20^d^	–6.94 ± 0.99^f^
PP	54^b^	51.5 ± 0.4	57.6 ± 4.7^d^	–68.9 ± 2.2^d^	–4.78 ± 0.15^d^	–0.70 ± 0.08^f^
PE	65^b^	65.6 ± 0.2	69.1 ± 0.2^d^	–71.3 ± 5.1^d^	–4.95 ± 0.35^d^	g

a Measurement obtained with TEM.

b Measurement obtained with DLS.

c Buffer containing 5 mM phosphate +5 mM SDS (pH = 8.9) and measured under low electric field conditions of *E* = 300 V/cm.

d Buffer containing 7.5% NH_3_ (pH = 11.9) and measured under low electric field conditions of *E* = 200 V/cm.

e Measured under high electric field conditions of *E* = 460 V/cm.

f Measured under high electric field conditions of *E* = 560 V/cm.

g Not measurable under current conditions.

h Conditions and equipment limitations did not allow for an accurate measurement of this hydrodynamic diameter.

### Instrumentation

Electrophoretic analyses were conducted with an Agilent Model 7100 (Santa Clara, CA) CE instrument equipped with a UV–vis spectrophotometric DAD detector. Electropherograms were treated with OpenLab software (version 3.6) from Agilent Technologies. Measurements of the hydrodynamic diameter and electrophoretic mobility were acquired on a Zetasizer ZS (Malvern Panalytical, Worcestershire, U.K.), operating in dynamic light scattering (DLS) and LDV modes, respectively. Measurements were carried out using a 4 mW He–Ne 633 nm laser module operating at 25 °C at an angle of 173° (back scattering), and results were analyzed using Malvern DTS 7.03 software. Bare fused silica capillaries supplied by Agilent Technologies were employed for all electrophoretic analyses. At the beginning of the day, prior to experimentation, the capillary was flushed for 5 min with 1 M NaOH, followed by water for 5 and 20 min with the background electrolyte, and then capillary integrity was verified by applying voltage during a short time period. Between runs, the capillary was preconditioned with 5 min of water and 5 min of buffer electrolyte prior to injection.

## Results and Discussion

### Electrophoretic Separation of PS Nanoparticles

Table 1 provides an overview of some particle features according to experimental conditions optimized throughout this work. At first, PS of different particle diameters (30, 60, 90, 200, and 300 nm) was used to perform preliminary tests by means of CE-DAD for size-based separation of nanoplastics. The former literature in this regard has been of help to establish optimal conditions for separation by CE.^20−22^Table 2 summarizes the main conditions for separation of PS particles by the proposed methodology.

**Table 2:** Optimized Operating Conditions for Separation of PS Particles by CE-DAD

parameter	optimal conditions
buffer composition	5 mM sodium phosphate + 5 mM SDS
buffer pH	8.9
applied voltage	30 kV
capillary length	65 cm
capillary i.d.	100 μm
cassette temperature	25 °C
injection time	5 s
injection pressure	50 mbar
wavelength	220 nm

**Figure 1:**
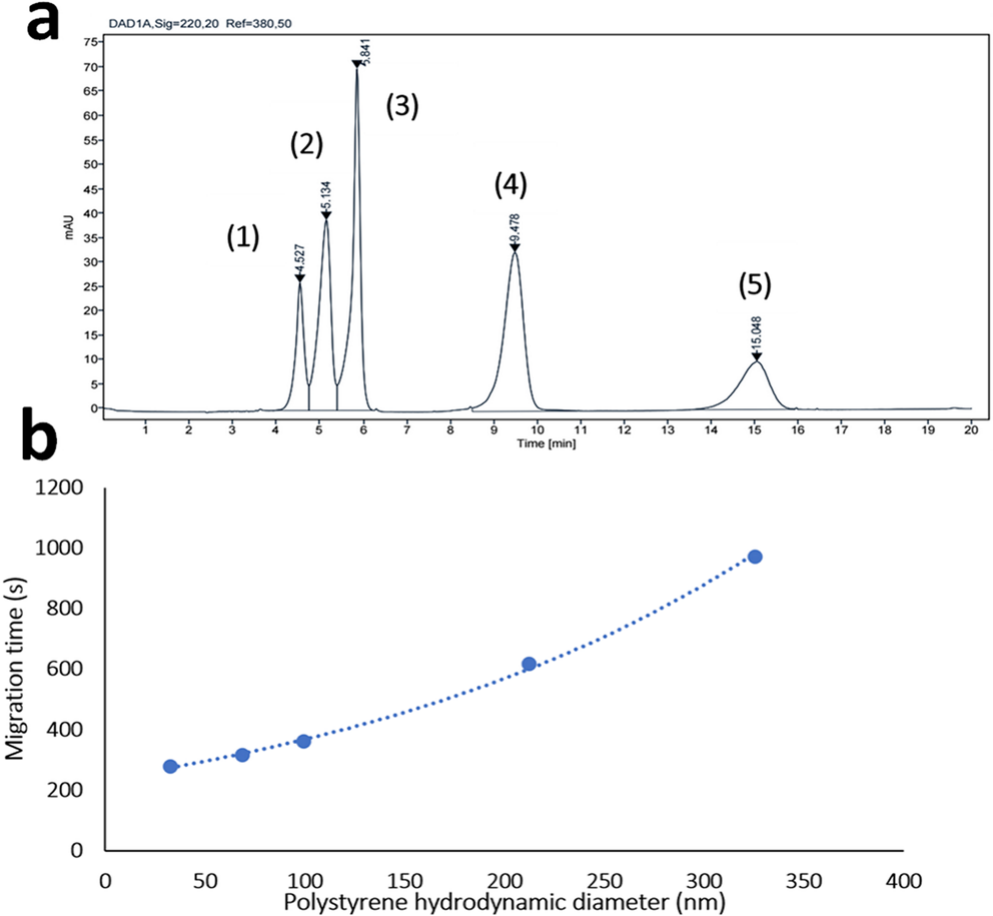
(a) Electropherogram acquired for PS NPs suspended in water by means of CE-DAD. Main conditions: fused silica capillary (65 cm length, 57 cm effective × 100 μm i.d.); cassette temperature 25 °C; hydrodynamic injection during 5 s at 50 mbar; applied voltage 30 kV; buffer 5 mM sodium phosphate dibasic with 5 mM sodium dodecyl disulfate at pH = 8.9; and detection at a 220 nm wavelength. Peak identification: (1) 31 nm PS 6.74 × 10^12^ particles mL^–1^; (2) 62 nm PS 8.43 × 10^11^ particles mL^–1^; (3) 92 nm PS 2.50 × 10^11^ particles mL^–1^; (4) 202 nm PS 2.27 × 10^10^ particles mL^–1^; and (5) 303 nm PS 6.74 × 10^09^ particles mL^–1^. (b) Plot of the migration time as a function of the particle hydrodynamic diameter under the optimal conditions of the proposed CE-DAD methodology. Error bars are included but are too small to be visible.

The first buffer component assayed was sodium borate (20 mM), with which no tall nor narrow peak was observed for 60 nm PS, regarded as a reference for UV signal monitoring. Therefore, alternative buffer components may favor a signal increase for PS or circumvent band broadening. Experiments with a neutral component, namely, ammonium acetate (5–10 mM), were conducted with the result of long runs (>15 min), thus ruling out this component as method quickness was pursued. Between tris(hydroxymethyl)aminomethane (20 mM) and sodium phosphate dibasic (2–10 mM), the former was discarded as a high-intensity current (∼60 μA) was observed during analysis in comparison with phosphate (<20 μA), and capillary integrity was another aspect to be preserved after several analyses. Sodium phosphate dibasic was selected because it induced sufficient charge on particles to exhibit a large absolute value of ζ-potential and electrophoretic mobility, thus favoring separation across the electric field. Further advantages of phosphate are its low absorptivity in the UV region and the fast electroosmotic flow provided in the electropherogram.^22^ However, with the sole presence of phosphate, no individual peak for each size was observed, given that some aggregates would be formed owing to closeness in size. By virtue of previous observations of the influence of sodium dodecyl sulfate (SDS) on peak resolution, this compound was added to the phosphate buffer to resolve five individual peaks. Indeed, the presence of this surfactant helped separate them all, with clear peaks on the electropherogram. Eventually, 5 mM sodium phosphate in conjunction with 5 mM SDS in an alkaline medium (pH = 8.9) was selected for an effective separation method of PS NPs in 15 min; through a bare fused silica capillary (57 cm effective length × 100 μm i.d.) and UV detection, signals were acquired at a 220 nm wavelength. The choice of the inner diameter was based on previous unsuccessful experiments with shorter inner diameters for PS particles larger than 200 nm when capillary clogging was experienced after injecting sizes larger than 200 nm, the capillary inner diameter being 50 μm. Injection time and pressure were also assessed, finding that the best compromise between sample introduction and peak resolution, avoiding band broadening, was at 50 mbar for 5 s. The applied voltage was tested between 20 and 30 kV, not affecting separation efficiency to a great extent and with minor differences regarding intensity current; therefore, the maximum value was selected. Hydrodynamic injection was chosen because it provides more reproducible results than electrokinetic injection.^23^Figure 1a shows a typical electropherogram for the separation of an aqueous mixture of five different PS sizes, from 30 to 300 nm, at a concentration range of 10^09^–10^12^ particles mL^–1^, with an increasing trend in migration time along with size, verified by prior separate injections for 30, 60, 90, 200, and 300 nm.

Peaks originating from each PS diameter are caused by UV-light absorption due to the molecular structure of the polymer, containing phenyl groups prone to charge-transfer interaction between electron-donating and electron-accepting groups on the same chain.^24^ Fundamentals described by Pyell for electrophoretic separation of NPs have been considered.^25^ To explain electrophoresis of charged spherical NPs, the following forces need to be considered: (i) the electrostatic force exerted on the charged particle, (ii) Stokes friction because of medium viscosity, (iii) electrophoretic retardation due to the electrostatic force exerted on the ion cloud, and (iv) the relaxation effect due to ion cloud distortion.^26^

**Figure 2:**
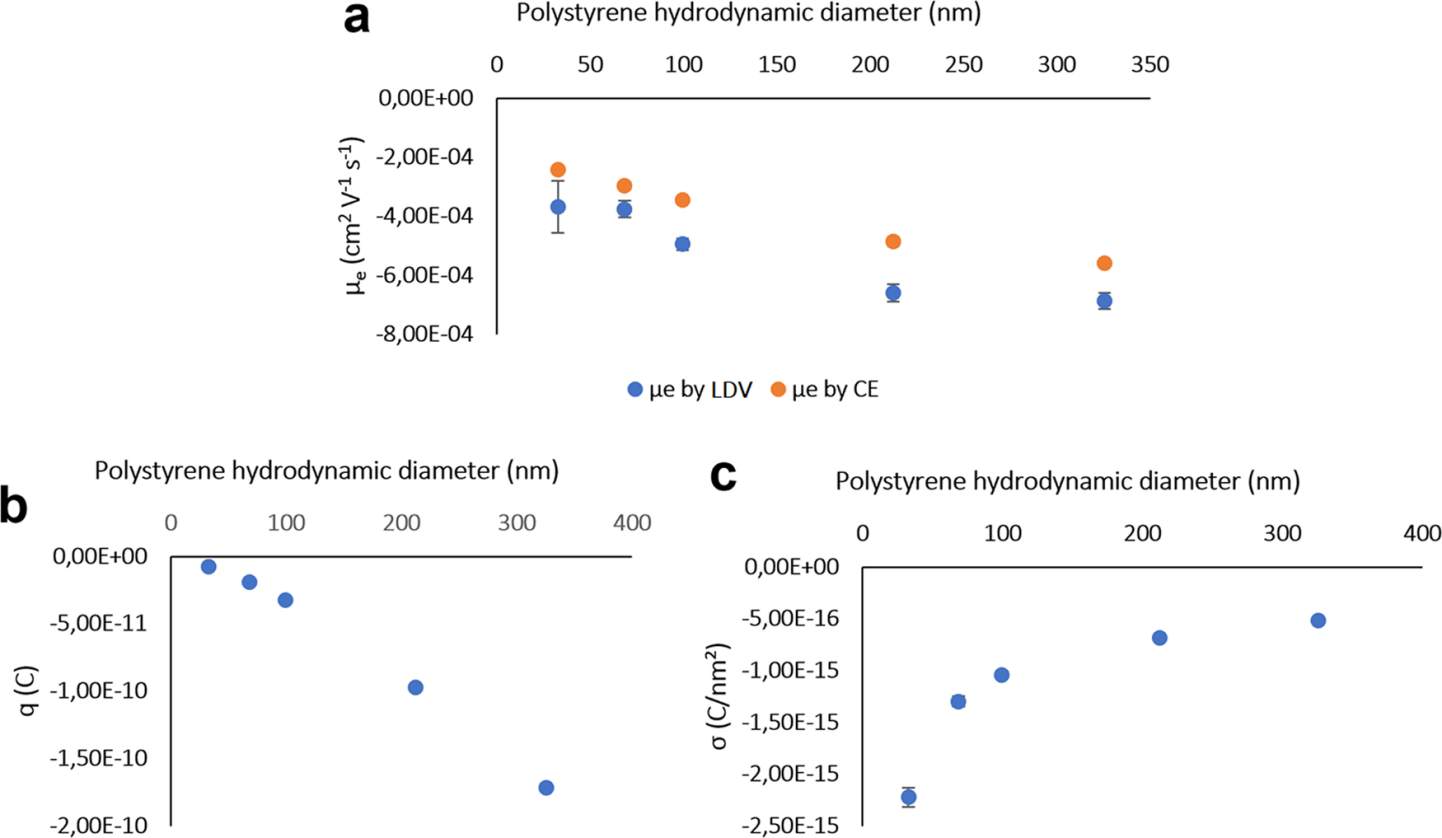
(a) Graphical correlation between the particle hydrodynamic diameter (acquired by DLS) in an aqueous suspension and values of electrophoretic mobility, both measured by LDV and calculated from migration times obtained in CE-DAD electropherograms for separation of PS NPs. (b) Size-dependent charge for PS NPs calculated from the effective electrophoretic mobility obtained from experimental CE migration times as a function of the hydrodynamic diameter. (c) Size-dependent SCD for PS NPs calculated from the effective electrophoretic mobility and particle charge for a spherical surface. Error bars are included, some of them being too small to be visible.

A mathematical correlation between size and migration time can be established for each PS NP (**e**). It can be observed that a linear trend exists for PS particles up to a 100 nm diameter, and beyond this dimension, the trend becomes exponential. A potential reason for this increase in the migration time for the larger particles may be EP_NL_ effects. In this perspective, larger particles are subjected to two electrophoretic effects, linear and nonlinear, both effects retarding particle migration as electrophoretic forces redirect particles toward the inlet. Consequently, larger particles are more prone to exhibiting stronger EP_NL_ effects than smaller ones.^9,16^ Another aspect to be considered is the likelihood of variation in population, surface charge, formation of aggregates, or interaction between particles and silanol groups across the capillary for larger sizes. An assessment of the relationship between the effective electrophoretic mobility and particle size is also included in this work; Figures 2a and 4a show these results for the PS and PMMA particles, respectively. Nonetheless, by means of this methodology, it may be possible to assign the particle diameter when migration time is known, this event being applicable to samples suspected to contain PS NPs of an unknown particle size distribution within this range.

The behavior of these differently sized particles has been investigated in terms of the mobilities of the two electrophoretic forces (linear and nonlinear). First, mobility for electroosmotic flow (μ_eof_) was calculated from eqs 1 and 2.
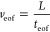
1
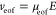
2in which *v*_eof_ is the velocity for electroosmotic flow, *L* is the total capillary length, *t*_eof_ is migration time for electroosmotic flow, and *E* is the electric field strength (*V*/*L*) applied across the capillary during the separation process. The overall particle velocity from CE experiments was determined employing eq 3
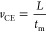
3where *t*_m_ is the migration time of the nanoparticle.^4^ The determination of the linear electrophoretic mobility (μ_e,l_) of the nanoparticles studied here was determined from CE experiments conducted at low electric field strengths to ensure that nonlinear effects were negligible. The conditions for these determinations are given in Table 1. The linear electrophoretic mobility data were estimated as follows
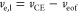
4
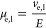
5where *v*_e,l_ and *v*_CE_ are the linear electrophoretic velocity and the CE velocity, respectively. The values for μ_e,l_ showed a downward trend from the smaller to the larger particles when calculating mobility based on experimental CE migration times, as depicted in Figure 2a. This phenomenon is in accordance with shorter migration times revealed by the smaller particles, while the larger particles show a higher magnitude on their negative electrophoretic mobility, resulting in longer migration times. Mobility values are considered negative as particles move electrophoretically as anions, although their net migration direction is to the cathode due to strong EOF.^27^ The linear electrophoretic mobility (μ_e,l_) is estimated with Helmholtz–Smoluchowski eq 6 or Henry’s eq 7, for which Ohshima developed an approximation eq 8 for *f*(κ*a*). These expressions are^28^
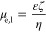
6
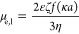
7
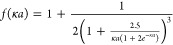
8where ε is the dielectric constant, ζ is ζ-potential for the particles, η is medium viscosity, and *f*(κ*a*) is the Henry function, where κ*a* is the relation between the particle radius and the double-layer thickness. The latter is typically termed 1/*k* (Debye length). The particle radius is represented as *a*. Electrophoretic determinations of ζ-potential are most commonly estimated in aqueous media and a moderate electrolyte concentration. In this scenario, *f*(κ*a*) is 1.5 and is referred to as the Smoluchowski approximation.

The next parameter characterized for all particles was their nonlinear electrophoretic velocity (*v*_e,nl_). It is important to note that there are two distinct regimes of nonlinear electrophoretic migration; the particles in this study followed the cubic regime, as described in eq 9.
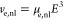
9where μ_e,nl_ is the mobility of the nonlinear electrophoresis velocity, which has been recently characterized for biological and synthetic particles, including viruses.^12,15,17,29−32^ The detailed explanation on how the appropriate regime for nonlinear electrophoretic migration was determined is included in the supporting material (eqs S1–S6 and **e**).^16,17,30,33^ CE experiments at higher electric field strengths (Table 1) were performed to ensure the presence of nonlinear electrophoresis, as this phenomenon is significant only at higher electric fields.^7^ The process for characterizing both the velocity (*v*_e,nl_) and mobility (μ_e,nl_) of the particles under nonlinear electrophoresis is described below^16,17,30,33^
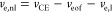
10
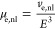
11where *v*_CE_ is the particle velocity from the CE experiments at high electric fields (see Table 1). The mobility of the nonlinear electrophoretic velocity experienced by the particles is reported in Table 1 for eight of the particles included in this study. This is the first reported experimental assessment of the effects of nonlinear electrophoresis on nanosized particles, and it was considered relevant to include these findings. More details on the effects of nonlinear electrophoresis on the nanoplastic particles studied here are included in the Supporting Information. Figures S3–S6 show the CE velocity of the particles studied here with and without considering the effects of nonlinear electrophoresis; the results clearly illustrate that nonlinear electrophoresis is significant and must be considered. Tables S4–S5 show that the velocity contribution of nonlinear electrophoresis can be as much as 59% of the contributions of linear electrophoresis (Table S5) at the maximum electric field employed in this work; this percentage value would be much higher at even higher electric fields. The values of the μ_e,nl_ obtained in this study are in accordance with the range of magnitude obtained for the virus, bacterial cells, and micron-sized particles.^12,15−17,29−32^

Calculations of particle charge and surface charge density (SCD) for each particle diameter have also been performed according to effective electrophoretic mobility obtained with CE experimental migration times and considering a spherical shape for the particles. Particle charge (*q*) was estimated from eq 12, considering the particle radius and medium viscosity, and SCD (σ) from eq 13, as a function of particle charge and the surface area for a sphere.
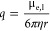
12
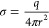
13where η is the viscosity of the medium and *r* is the particle radius. Figure 2b shows the distribution of charge according to the hydrodynamic radius of PS particles. Figure 2c shows the distribution of SCD according to the particle diameter. It was found that the negative SCD magnitude follows a decreasing trend when particle size increases, in a similar way to that reported in the literature for spherical NPs.^34^ Similar effects have also been recently observed in microparticles.^16^ A sharp drop in SCD can be observed for the smaller particles, and for larger particles beyond 300 nm, SCD may reach a plateau and become almost independent of size. This hypothesis may be in accordance with previous work on PS separation, as it was found that particles with a diameter beyond 300 nm show small differences in electrophoretic mobilities and provide a poor peak resolution in electropherograms.^22^

Regarding analytical figures of merit, the detection limit was found to be in the concentration region of 10^11^ particles mL^–1^ (*n* = 3, 95%) for the smaller sizes, while repeatability obtained for the migration time and peak area was lower than 4 and 10%, respectively. Table S1 summarizes the main peak parameters from electropherograms and precision figures for separation of PS NPs.

### Electrophoretic Separation of PMMA Nanoparticles

The performance of the aforementioned methodology was assessed for other plastic NPs. It was found that a phosphate buffer in combination with SDS was not suitable for the separation of PMMA NPs, as the signal shown on UV was poor and no separation would occur. Alternatively, to reach stronger alkaline conditions, an ammonium hydroxide buffer was tested, and it was observed that these particles could be successfully separated by their different size in this ammonia-bearing electrolyte at pH = 11.9 in less than 10 min, signals acquired at a 220 nm wavelength. This is the first time that CE is demonstrated to separate these type of particles. Parameter study and optimization were carried out in a similar way to the approach developed for PS particles. Buffer composition was optimized between 2–10 mM phosphate dibasic with 5–10 mM SDS and 1–10% ammonium hydroxide, a compromise being found with 7.5% ammonium hydroxide according to its ionic strength. Buffer pH was 11.9, separation voltage was set at 28 kV after attempts within the range 20–30 kV, capillary dimensions were chosen between 40–70 cm and 50–100 μm for total length and i.d., respectively, opting for a 50 cm length and 75 μm as the best i.d., the cassette temperature was fixed at 25 °C, and the more convenient wavelength selected for DAD acquisition was 220 nm. Hydrodynamic injection was also preferred for PMMA separation, selecting 50 mbar and 5 s for injection pressure and injection time, respectively.

**Figure 3:**
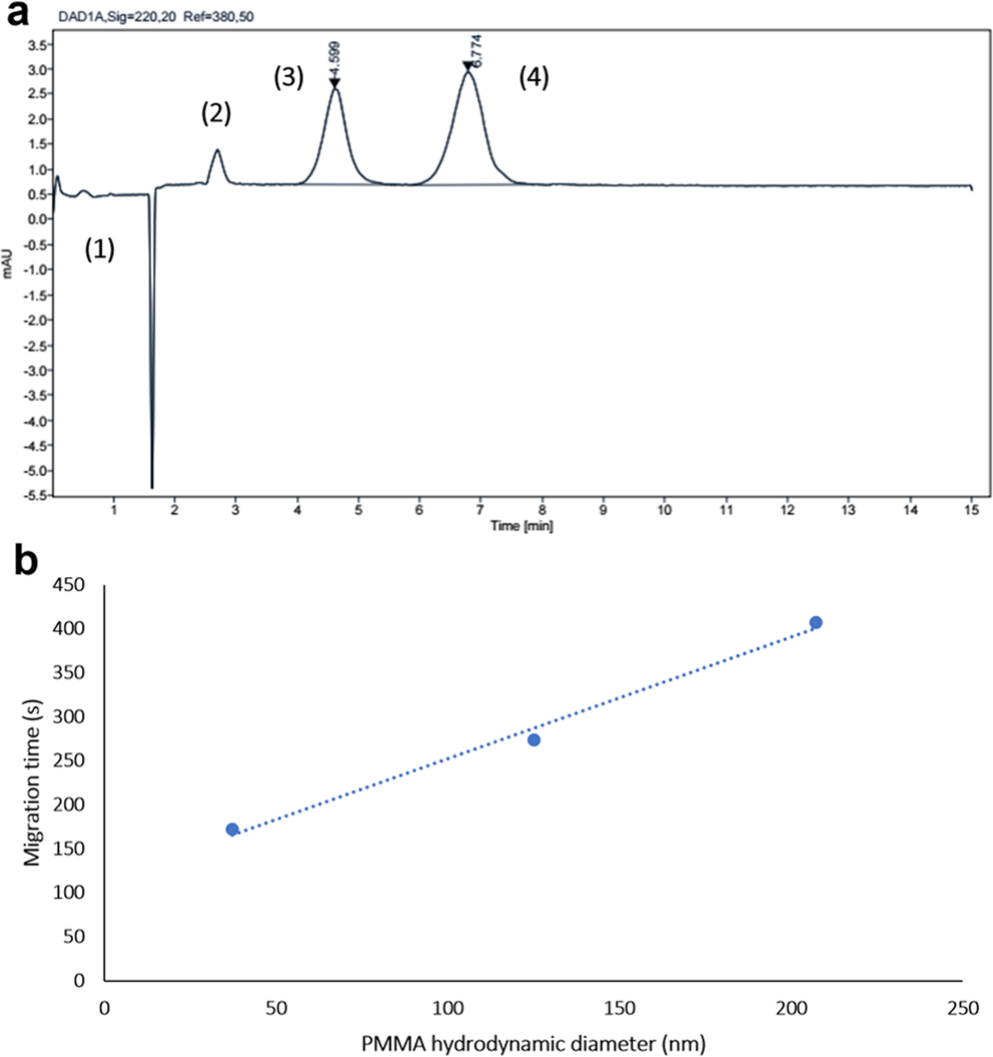
(a) Electropherogram acquired for PMMA NPs suspended in water by means of CE-DAD. Main conditions: fused silica capillary (50 cm length, 42 cm effective × 75-μm i.d.), cassette temperature 25 °C, hydrodynamic injection during 5 s at 50 mbar, applied voltage 28 kV, buffer 7.5% ammonium hydroxide at pH = 11.9, and detection at a 220 nm wavelength. Peak identification: (1) EOF, (2) 38 nm PMMA 1.30 × 10^12^ particles mL^–1^, (3) 103 nm PMMA 1.62 × 10^11^ particles mL^–1^, and (4) 219 nm PMMA 2.02 × 10^10^ particles mL^–1^. (b) Mathematical correlation between the particle hydrodynamic diameter in the water suspension and migration time under the optimal conditions of the proposed CE-DAD methodology for separation of PMMA NPs. Error bars are included but are too small to be visible.

A typical electropherogram for the separation of an aqueous mixture of three different sizes of PMMA particles is shown in Figure 3a, with the concentration range being 10^10^–10^12^ particles mL^–1^. Peaks exhibited by each PMMA diameter are suspected to be provoked by UV-light absorption of the carbonyl group present in the molecular structure; therefore, this polymer is also prone to showing absorbance. However, according to the signal on the electropherogram, this absorption phenomenon occurs to a lesser extent than PS when particles are detected by DAD. The promising feature of this approach is undoubted, as there are no previous studies on the separation of PMMA by means of CE-based methodologies.

When the particle diameter correlates with migration time, it can be observed that a linear trend exists between the three sizes subject to study. In this case, linearity was found to reach larger sizes, up to 200 nm, as Figure 3b depicts. In an analogous way to that reported with PS, this methodology may also allows for assigning the particle diameter based on migration times in the electropherogram, with applicability to samples suspected to bear PMMA NPs of an unknown size distribution within this range.

**Figure 4:**
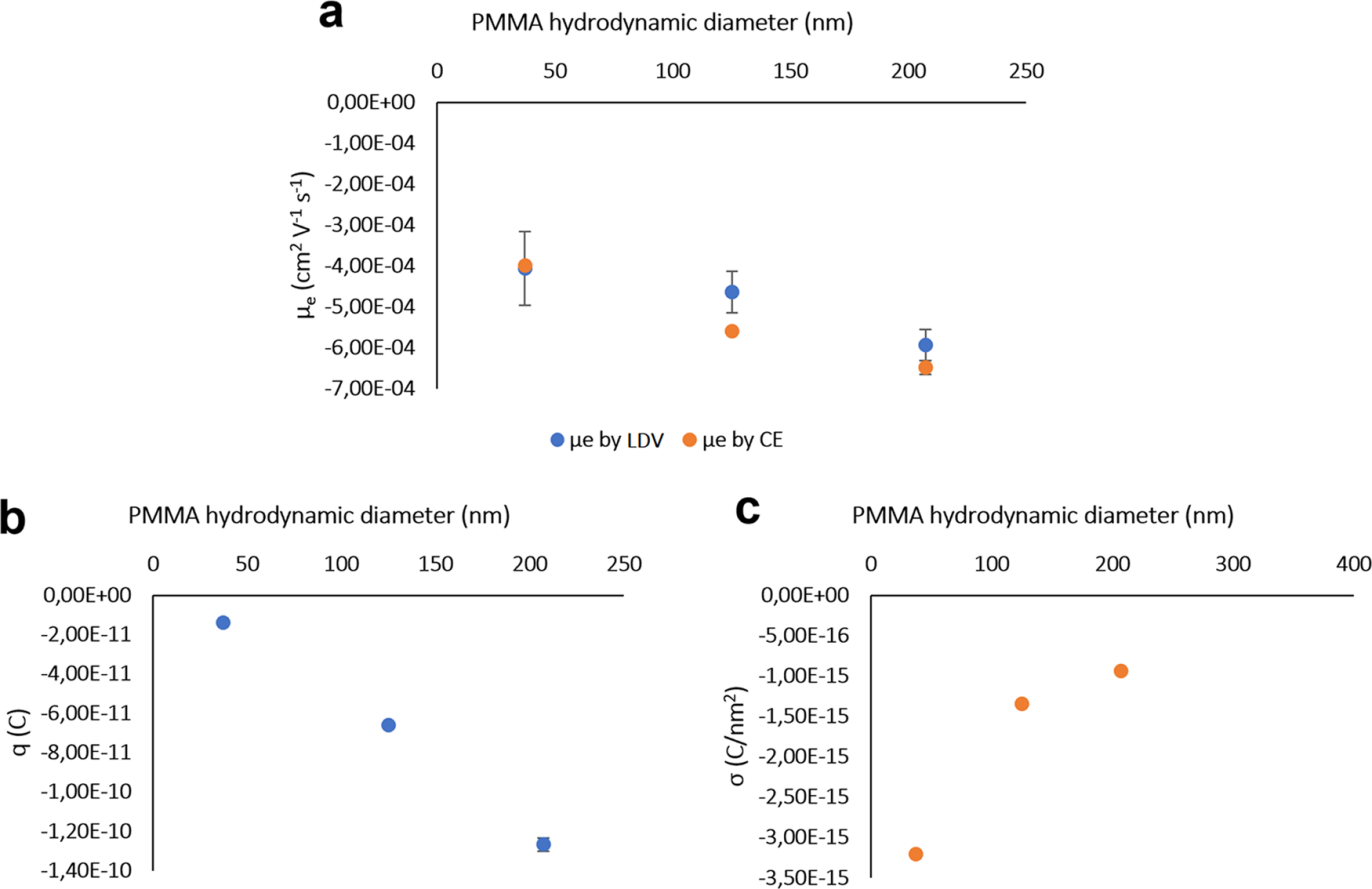
(a) Graphical correlation between the particle hydrodynamic diameter (acquired by DLS) in the aqueous suspension and values of electrophoretic mobility, both measured by LDV and calculated from migration times obtained in CE-DAD electropherograms for the separation of PMMA NPs. (b) Size-dependent charge for PMMA NPs calculated from effective electrophoretic mobility and the hydrodynamic radius. (c) Size-dependent SCD for PMMA NPs calculated from effective electrophoretic mobility, particle charge, and hydrodynamic radius for a spherical surface. Error bars are included, some of them being too small to be visible.

Values for the electrophoretic mobility, as shown in Figure 4a, also exhibited a downward trend from smaller to larger particles. The effective electrophoretic mobility was calculated based on experimental migration times from electropherograms, making use of eqs 1–5.

The results of the characterization of the migration of PMMA under nonlinear electrophoretic effects are reported in Table 1. Also, as was done with PS particles, the Supporting Information contains Figure S4 that shows a plot comparing the particle CE migration velocity with and without the linear electrophoresis effect, where it is clear that nonlinear electrophoresis has a major impact on particle migration. These results are further supported by Table S5, which shows that the nonlinear electrophoretic velocity can be up 59% of the magnitude of the linear electrophoretic velocity. These findings illustrate that the nonlinear electrophoretic effect must be considered to accurately predict particle velocity and migration times in a CE system under high electric field magnitudes.

The behavior of PMMA particles in terms of *q* and SCD was similar to that of PS particles. Figure 4b shows the distribution of *q*, and Figure 4c shows the distribution of SCD versus particle hydrodynamic diameter in both cases. The trend in *q*, estimated with eq 12, is a negative straight line. Figures for SCD were estimated from *q* and considering spherical shape with eq 13. The same decreasing trend in the SCD magnitude was observed as particle size increased. The sharp drop in SCD would occur for the smaller particles (<100 nm), and for larger particles exceeding 200 nm, SCD may reach a plateau and become independent of size. This may also lead to a poor separation degree or even no resolution for larger particle sizes, as demonstrated in the literature for PS. There is no study on electrophoretic behavior of PMMA and its separation by CE; thus, this is the first approach to a better understanding of particle mobility for this polymer, opening an unexplored highway for a deeper insight into this or other kinds of polymeric analytes.

Analytical figures of merit were similar to those provided for PS. Sensitivity was not improved in relation to the previous methodology, as detection limit was found to be at 5 × 10^11^ particles mL^–1^ (*n* = 3, 95%) for the smaller sizes, while repeatability obtained for the migration time and peak area was lower than 10% in all cases. The first main pitfall of the aforementioned approaches is the lack of sensitivity that may impact the analysis of real-life samples, expected to contain lower amounts of nanoplastics. This drawback is to be circumvented by preconcentration approaches or CE hyphenation with more sensitive detection devices. Table S2 summarizes the main peak parameters from electropherograms and precision figures for the separation of PMMA NPs.

## Conclusions

Considering the achievements described in this paper, it is likely to outline that, on the one hand, CE is a valuable technique for the separation and detection of nanoplastics by the particle diameter, considering surface charge density. The electrophoretic behavior of differently sized PS particles has been studied, demonstrating that a separation dependent on size is feasible with CE-DAD, making use of a phosphate buffer together with SDS in alkaline conditions. The smaller particles migrated sooner than the larger particles; these observations were corroborated by LDV measurements of effective electrophoretic mobility and compared with experimental mobility obtained from migration times. Calculations of SCD also showed this parameter as size-dependent, even if only within the size range studied. A consistent correlation between the particle diameter and migration time was also observed, with implications in unknown nanoplastic-bearing samples within the size range subject to study. Additionally, a CE analytical approach to the separation of PMMA, PP, and PE NPs has been reported for the first time, highlighting the promising feature of this technique regarding polymer analysis. In this case, an ammonium hydroxide buffer was indicated to reach stronger alkaline conditions and provoke the desired interaction between the electrolyte and particles to be discriminated. Similar observations were confirmed regarding size-based separation and electrophoretic mobility for both CE and LDV measurements as well as SCD calculations. Repeatability was acceptable in all cases, no higher than 10% for both the migration time and peak area, and method quickness might be of interest. Furthermore, a size detection limit does not seem to exist for these polymeric particles, and it may be achievable to discriminate nanoplastics in the region of tens of nanometers even if they are very close in size. Additionally, the effects of nonlinear electrophoretic migration, a phenomenon observed in the migration of microparticles in similar systems, were also quantified in this study for eight out of ten nanoplastic particles assayed, making this work the first report on the effects of nonlinear electrophoresis migration of nanoplastic particles in a CE system.

The sensitivity for the analytes subject to study is low, in the concentration region of 10^11^ particles mL^–1^, and the applicability of CE-UV analysis to actual samples is difficult to be implemented. Molecules prone to absorbing UV light or overlapped peaks for nanoplastic mixtures are certain obstacles to the analysis of actual samples with CE-UV. First, preconcentration strategies (filtration, centrifugation) are strongly encouraged in order to reach actual nanoplastic levels in real-life samples, together with CE hyphenation with state-of-the-art detection devices (e.g., inductively coupled plasma mass spectrometry (ICP-MS), liquid chromatography-MS (LC-MS), MS) to circumvent sensitivity limitations and gather knowledge about polymers present in the samples. Furthermore, sizes subject to analysis may be constrained to figures such as 300 nm, and larger sizes may also cause capillary clogging or peak tailing due to undesired interactions across the capillary. A more universal approach for plastic particle detection and sensitivity enhancement needs to be pursued by CE-based approaches. These suggestions are further steps to be given in the short term with the aim of optimizing CE-based analytical methods with implications in societal concerns.

## Supporting Information Available

Assessment of electrophoretic mobility determination, further experimental details and analytical parameters, electrophoretic separation of nanoplastic mixtures, assessment of contributions to particle velocity from electroosmotic flow, and linear and nonlinear electrophoresis (PDF)
