# Supplementary material for: Capillary Electrophoresis as a Complementary Analytical Tool for the Separation and Detection of Nanoplastic Particles
Source: Anal Chem. 2024 Apr 30;96(19):7706–13. doi: 10.1021/acs.analchem.4c00822 (PMC11099890; doi:10.1021/acs.analchem.4c00822)
Supplement: Supplementary file 1 — ac4c00822_si_001.pdf [file ac4c00822_si_001.pdf]

Supporting Information

Capillary Electrophoresis as a Complementary Analytical Tool for the Separation and Detection of Nanoplastic Particles

Carlos Adelantado[1,*], Blanca H. Lapizco-Encinas[2], Jan Jordens[1], Stefan Voorspoels[1], Milica Velimirovic[1], and Kristof Tirez[1]

[1]Flemish Institute for Technological Research (VITO), Boeretang 200, 2400 Mol, Belgium

[2]Microscale Bioseparations Laboratory and Biomedical Engineering Department, Rochester Institute of Technology, 160 Lomb Memorial Drive, Rochester, New York 14623, United States

*Corresponding author: Carlos Adelantado – Separation and Conversion Technology, Flemish Institute for Technological Research (VITO), Boeretang 200, 2400 Mol (Belgium); (orcid.org/0000-0003-1360-3110); Email: carlos.adelantadosanchez@vito.be

Table of Content

INFORMATION ON POTENTIAL USE OF LASER DOPPLER VELOCIMETRY (LDV) IN THE ASSESSMENT OF ELECTROPHORETIC MOBILITY S2

INFORMATION ON THE REAGENTS EMPLOYED TO PREPARE BUFFER SOLUTIONS S2

PEAK PARAMETERS FROM ELECTROPHEROGRAMS AND PRECISION FIGURES FOR SEPARATION OF PLASTIC NANOPARTICLES: CONTAINS TABLES S1-S2 .... S2

ELECTROPHORETIC SEPARATION OF OTHER POLYMERIC NANOPARTICLES: CONTAINS FIGURES S1-S2 .... S3

IDENTIFICATION OF THE APPROPRIATE REGIME OF ELECTROPHORESIS MIGRATION: CONTAINS TABLE S3 .... S5

ASSESSMENT OF THE CONTRIBUTIONS TO PARTICLE OVERALL VELOCITY FROM ELECTROOSMOTIC FLOW, LINEAR ELECTROPHORESIS, AND NONLINEAR ELECTROPHORESIS: CONTAINS FIGURES S3-S6 AND TABLES S4-S5 .... S7

REFERENCES S13

Information on potential use of laser Doppler velocimetry (LDV) in the assessment of electrophoretic mobility

Apart from CE, LDV is an established method for determining the electrophoretic mobility of particles in life sciences, and has been incorporated in commercial devices.[1–3] Electrophoretic mobility acquired by LDV measurements might be employed to characterise nanoparticle migration under linear electrophoresis, even though in this study CE at low electric fields conditions was preferred to calculate linear electrophoretic mobility, so as to conduct the entire research project on a sole instrument.

Information on the reagents employed to prepare buffer solutions

Sodium hydroxide aqueous solution (1 M) and buffer solution containing 20 mM sodium borate were provided by Agilent Technologies (Santa Clara, CA, USA). Ammonium acetate (≥98%), tris(hydroxymethyl)aminomethane (≥99.9%), and sodium phosphate dibasic (≥99.0%) were obtained from Sigma Aldrich (MO, USA). Sodium dodecyl sulphate (≥99%) was purchased from Fluka (Switzerland). Ammonia solution (25%) was supplied by Sigma Aldrich (Germany). Ultra-pure water (18.2 MΩ cm) was dispensed from a Milli-Q system (Millipore, Burlington, MA, USA).

Peak parameters from electropherograms and precision figures for separation of plastic nanoparticles: contains Tables S1-S2

Table S1. Peak parameters and precision figures for separation of PS nanoparticles from 30 to 300 nm by the proposed CE-DAD method for the said particles.

PS particle diameter (nm)	Peak area[a]	Migration time[a] (s)	Repeatability, RSD[a] (%)	
			Peak area	Migration time
31	281,4	271,6	2,7	0,3

62	278,6	308,0	9,8	1,7
92	263,2	350,5	6,9	1,4
202	188,1	568,7	3,8	1,6
303	272,8	902,9	7,6	3,9

[a] For PS particle concentrations from $6{,}7 \times 10^{09}$ particles mL^{-1} to $6{,}7 \times 10^{12}$ particles mL^{-1} (n = 3).

Table S2. Peak parameters and precision figures for separation of PMMA nanoparticles from 50 to 200 nm by the proposed CE-DAD method for the said particles.

PMMA particle diameter (nm)	Peak area[a]	Migration time[a] (s)	Repeatability, RSD[a] (%)	
			Peak area	Migration time
38	27,6	172,3	5,6	0,7
103	211,9	273,4	4,4	0,4
219	416,0	407,6	8,7	9,7

[a] For PMMA particle concentrations from $6{,}7 \times 10^{10}$ particles mL^{-1} to $4{,}3 \times 10^{12}$ particles mL^{-1} (n = 3).

Electrophoretic separation of other polymeric nanoparticles: contains Figures S1-S2

In view of the effectiveness of ammonium hydroxide as a background electrolyte to interact selectively with PMMA particles, the same conditions were assayed for PE and PP particles. Previously, it was observed that the phosphate medium for polystyrene separation was only suitable for these particles. Additionally, it was foreseen that no UV signal would be acquired for PE and PP, as they are polyolefins not prone to absorbing UV-visible light. Unexpectedly, after injection of PP (54-nm particle diameter) and PE (65-nm particle diameter) nanoparticles, it was found that separate peaks can be obtained under the optimised conditions for PMMA particle resolution. Fig. S1a and Fig. S1b show electropherograms for separation of PP and PE particles at a concentration of 10^{12} particles mL^{-1}, signals being acquired at 254-nm wavelength.

The case of these polymers is not similar to the previous studies, given that there are no functional groups and no chromophores in the structure of PP and PE. Therefore, no UV light absorption and no peak on electropherograms was expected. Nevertheless, signal on DAD for both polymers was clearly spotted. It is likely to expect that these particles may be blocking UV light when reaching the detection window, then provoking a signal change and peak appearance. This hypothesis may also explain that a large concentration of these particles was necessary to promote such a peak, whose intensity is lower than that of the previous cases. The interest of this finding is the possibility to analyse other structures of polymers, even though they are not absorbing UV light.

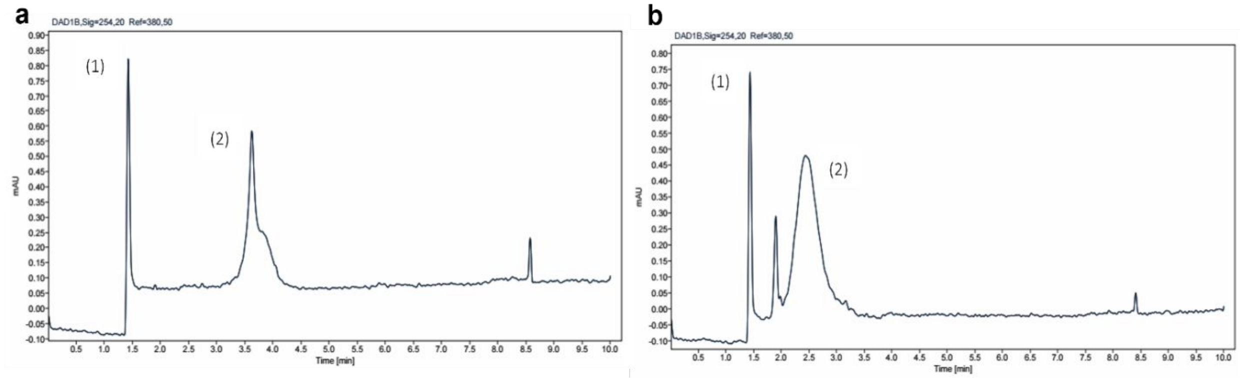

Figure S1. Electropherograms acquired for plastic nanoparticles suspended in water by means of CE-DAD. Main conditions: fused silica capillary (50 cm length, 42 cm effective × 75-µm i.d.); cassette temperature 25ºC; hydrodynamic injection during 5 s at 50 mbar; applied voltage 28 kV; buffer 7.5% ammonium hydroxide at pH=11.9; detection at 254-nm wavelength. (a) Peak identification: (1) EOF: (2) 54-nm PP 6,60x10^{12} particles mL^{-1}. (b) Peak identification: (1) EOF; (2) 65-nm PE 3,87x10^{12} particles mL^{-1}.

In order to present an initial approach simulating real-case scenarios, several mixtures of polymers were injected in the CE system under the aforementioned conditions for separation of PMMA, PP and PE particles. The electropherogram exhibited on Fig. S2a is an attempt to resolve the three sizes of PMMA previously analysed, together with 54-nm PP and 65-nm PE, in a concentration range from 10^{10} to 10^{12} particles mL^{-1}.

The difficulty in assigning peaks is a limitation of the approach. Even if the two peaks in the region from 3 to 5 min for migration time seem to correspond to 100-nm and 200-nm PMMA, an earlier region with overlapped peaks after EOF is also observed. The closeness in size for 50-nm PMMA, 54-nm PP and 65-nm PE may be an explanation for the poor resolution of these peaks, as three separated signals are initially expected. To clarify this scenario, LDV measurements for effective electrophoretic mobility were separately acquired for the five particle diameters, differences between values being shown on Fig. S2b. It was observed that mobility figures are similar for PP, PE and the smaller PMMA diameter, thus these minor differences in migration features may explain the broad signal for the three species.

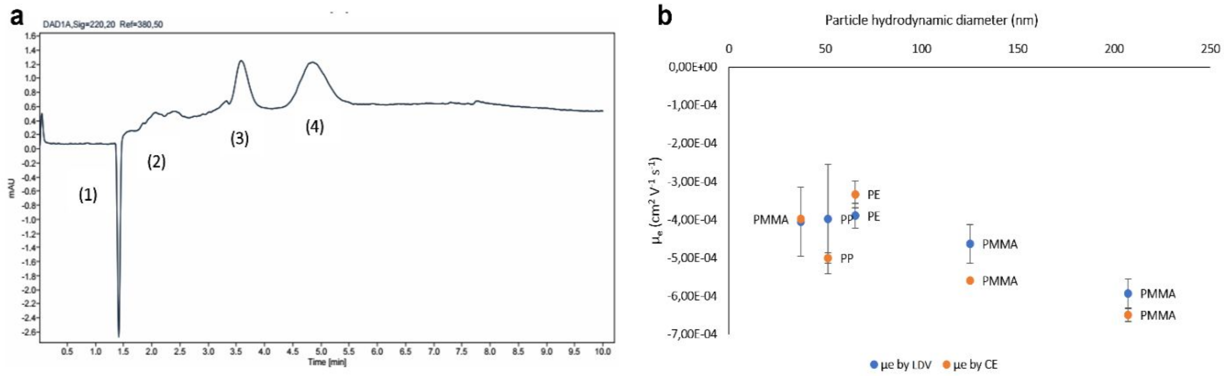

Figure S2. (a) Electropherogram acquired for a mixture of PMMA, PP and PE nanoparticles suspended in water by means of CE-DAD. Main conditions: fused silica capillary (50 cm length, 42 cm effective × 75-μm i.d.); cassette temperature 25ºC; hydrodynamic injection during 5 s at 50 mbar; applied voltage 28 kV; buffer 7.5% ammonium hydroxide at pH=11.9; detection at 220-nm wavelength. Peak identification: (1) EOF; (2) overlaid peak comprising 65-nm PE 3,87x10^{11} particles mL^{-1}, 54-nm PP 6,60x10^{12} particles mL^{-1} and 38-nm PMMA 6,50x10^{11} particles mL^{-1}; (3) 103-nm PMMA 1,62x10^{11} particles mL^{-1}; (4) 219-nm PMMA 1,62x10^{11} particles mL^{-1}. (b) Effective electrophoretic mobilities acquired separately by LDV and calculated from CE migration times under the aforementioned conditions for separation.

Nevertheless, the positive aspect is a consistent correlation between migration time and particle diameter, together with the agreement between LDV and CE effective mobilities, an example of this fact exhibited for PS and PMMA. Particles below 100 nm seem to migrate within a certain region close to the electroosmotic flow, regardless of their structure. This reasoning may lead to the conclusion that an unknown sample containing particles in a size distribution within the size range assayed in this work may be analysed by CE, their peaks being assigned to a certain particle diameter based on migration time.

Identification of the appropriate regime of electrophoresis migration: contains Table S3

To identify the appropriate regime or dependence with the electric field of particle migration under electrophoresis, it is necessary to rely on three dimensionless parameters: the dimensionless

applied field magnitude (β), the Peclet (*Pe*) and the Dukhin (*Du*) numbers, which are described by the following expressions:[1–4]

$$\beta = \frac{Ea}{\varphi} \quad \text{(S1)}$$

$$Pe = \frac{a|v_{EP}|}{D} \quad \text{(S2)}$$

$$Du = \frac{K^{\sigma}}{K_m a} \quad \text{(S3)}$$

where E is the magnitude of electric field, a is the particle radius, φ is the thermal voltage (~25 mV), v_{EP} is the magnitude of the electrophoretic velocity (linear and nonlinear contributions), D is the diffusion coefficient, K^{σ} and K^{m} are the surface and bulk conductivity of the medium, respectively.

To provide a brief explanation on how electrophoretic migration is characterized as linear or nonlinear employing the dimensionless parameters in Equations (S1-S3), it is important to first describe, concentration polarization. When a particle is exposed to an electric field, the electrical double layer (EDL) that surrounds the particles is deformed, the concentration of the ions in the EDL is no longer homogenous. This phenomenon is called concentration polarization.[4–7] Under low to moderate electric fields, the concentration polarization is low, and diffusion dominates ion transport within the EDL. However, as the electric field increases, convective transport increases and starts to affect the diffusion process, increasing the concentration polarization and giving rise to induce-charge electrokinetics due to changes in the surface conductance. Nonlinear electrophoresis, also called electrophoresis of the second kind, depends on the bulk charge that surrounds the particle which is affected by concentration polarization. As mentioned, concentration polarization depends on the electric filed magnitude, which is described by the dimensionless applied electric field (β), the convective transport of the bulk charge is described by the Peclet number (*Pe*) and the ratio of the surface conductivity to that of the medium is described by the Dukhin number (Du).[4–7]

There are three distinct regimes of the dependency the electrophoretic velocity with the electric field, these regimes are:[55–7]

- **<u>Weak field regime</u>**, this is the linear dependence (**E**[1]), which occurs under these conditions: $\beta <<$ 1, *Pe* << 1, Du ~ 0. The electrophoretic velocity is described by:

$$v_{e,l} = \mu_{e,l}\, E \quad \text{(S4)}$$

- **Moderate field regime**, this is the cubic dependence (**E**^{3}), which occurs under these conditions: $\beta \leq 1$, *Pe* << 1, Du arbitrary.

  $v_{e,nl}^{(3)} = \mu_{e,nl}^{(3)} E^3$ (S5)

- **Strong field regime**, this is the linear dependence (**E**$^{3/2}$), which occurs under these conditions: $\beta > 1$, *Pe* >> 1, Du << 1.

  $v_{e,nl}^{(3/2)} = \mu_{e,nl}^{(3/2)} E^{3/2}$ (S6)

Under the experimental conditions employed in this work, in particular the small size of the particle, the nanoparticles can only reach the moderate field regime (as β never exceed the value of 1), which means that the EP_{NL} velocity has a cubic dependence with the electric field ($v_{e,nl}^{(3)} = \mu_{e,nl}^{(3)} E^3$). The values of Beta and Peclet number are include in **Table S3**.

Table S3. Moderate field regime parameters illustrating the cubic dependence (E^3), which occurs with $\beta \leq 1$, *Pe* << 1, Du arbitrary.

Particle	Diameter by supplier in nm	β	Pe	Du
PS	31	0,030	0,014	Not needed, arbitrary
PS	62	0,063	0,032	Not needed, arbitrary
PS	92	0,092	0,052	Not needed, arbitrary
PS	202	0,195	0,129	Not needed, arbitrary
PMMA	38	0,037	0,016	Not needed, arbitrary
PMMA	103	0,077	0,048	Not needed, arbitrary
PMMA	219	0,112	0,114	Not needed,

				arbitrary
PP	54	0,238	0,032	Not needed, arbitrary

Assessment of the contributions to particle overall velocity from electroosmotic flow, linear electrophoresis, and nonlinear electrophoresis: contains Figures S3-S6 and Tables S4-S5

To assess the contribution of nonlinear electrophoresis to the overall particle electromigration velocity it was necessary to analyse the contribution of each one of the three electrokinetic phenomena present in the system: electroosmotic flow, linear electrophoresis, and nonlinear electrophoresis.

It is important to note, that CE experiments were performed at low electric fields and also at high electric fields. Experiments at low electric field were employed to determine the linear electrophoretic mobility ($\mu_{e,l}$), since at low electric field the contributions of nonlinear electrophoresis to particle migration are negligible. High electric fields are required for nonlinear electrophoresis to have an effect on particle migration. Thus, experiments at E= 300 V/cm and E=200 V/cm, were performed with the PS particles and the PMMA-PP-PE particles, respectively (see **Table 1**). After the liner electrophoretic mobility had been determined, employing the results from the CE experiments carried out a low electric field. At second set of experiments was carried out the higher electric field of at E= 460 V/cm and E=560 V/cm, were performed with the PS particles and the PMMA-PP-PE particles, respectively (see **Table 1**). This second set of experiments allowed the determination of the nonlinear electrophoretic mobility ($\mu_{e,nl}$).

To illustrate the effect on nonlinear electrophoresis, **Figure S3** includes a plot of the CE velocity of the PS nanoparticles studied in this work as a function of the electric field. For each particle, two distinct CE velocities are included. CE velocities (labeled as linear and represented by dotted lines) only consider the contributions of EOF and linear electrophoresis ($v_{CE,\,linear} = v_{eof} + v_{e,l}$)

and CE velocities which (labeled as real and represented by continuous lines) consider the contribution of nonlinear electrophoresis ($v_{CE,\,linear} = v_{eof} + v_{e,l} + v_{e,nl}$). As can be seen from **Figure S3**, nonlinear electrophoresis has an important impact of particle CE velocity, which in turn impacts particle migration time. The effects of nonlinear electrophoresis decrease the overall CE velocity of the particle and increase particle migration time, that is, particles are more retarded due to nonlinear electrophoresis effects. To further illustrate the effects of nonlinear electrophoresis, let's analyse for example, the 31 nm PS particle (shown in red color in **Figure S3**), which should reach a CE velocity of 0.39 cm/s at E=800 V/cm under the current operating conditions if nonlinear electrophoresis is neglected. However, in reality, the CE velocity of 31 nm PS particle is only 0.24 cm/s at E=800 V/cm. This is an excellent example of the impact of nonlinear electrophoresis on particle velocity and migration.

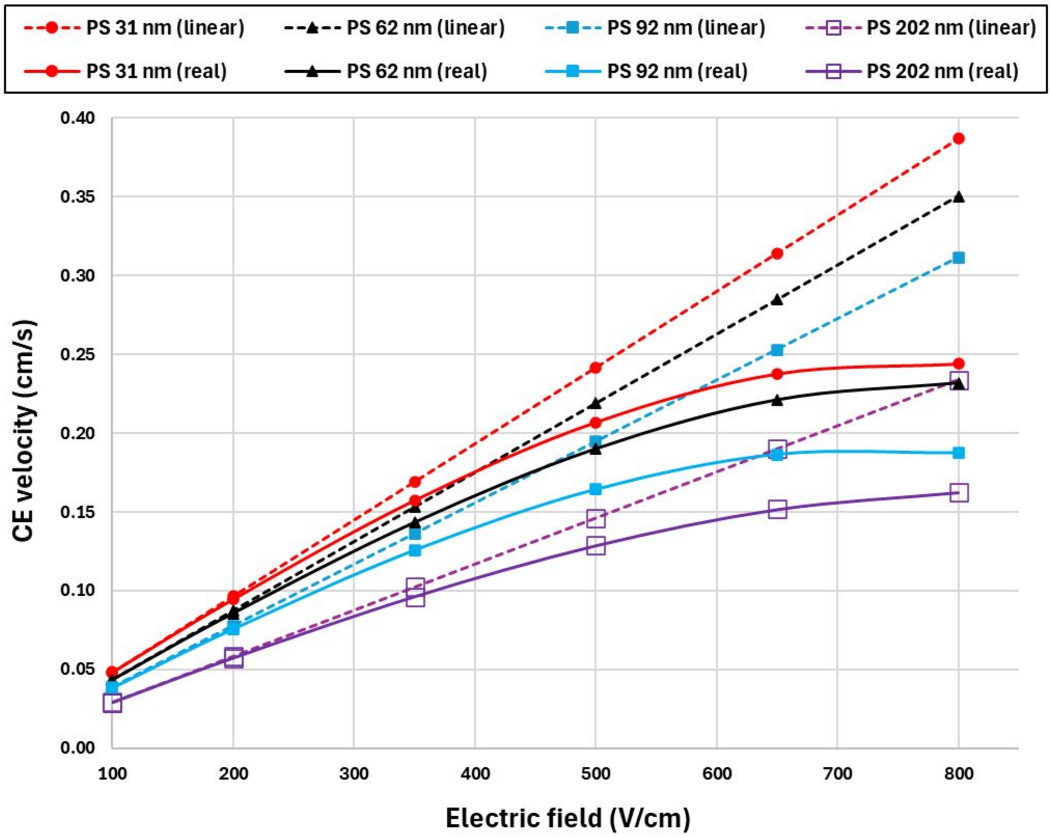

Figure S3. (a) Plot of CE velocity as a function of the electric field for four types of PS nanoparticles. It was not possible to characterize the nonlinear electrophoretic migration of the PS 303 nm particles. CE linear velocity, represented as dotted lines, neglects the effect of nonlinear electrophoresis, while CE real velocity,

represented as continuous lines considers the effects of nonlinear electrophoresis. To aid visualization, the following markers were employed: filled circle for the PS 31 nm particles, triangle for the PS 62 nm particles, filled square for the PS 92 nm particle and large unfilled square for the PS 202 nm particles.

To further highlight the importance of nonlinear electrophoresis, a plot of the electrophoretic velocity as a function of the electric field was also prepared, these results are in **Figure S4**. Similar to **Figure S3**, the plot contains four PS nanoparticles, where the linear electrophoretic velocity estimations ($v_{EP,\,linear} = v_{ep,l}$) without considering nonlinear electrophoresis are represented in dotted lines (labeled as linear) and the real electrophoretic velocity estimations ($v_{EP,\,linear} = v_{ep,l} + v_{ep,nl}$) considering the effects of nonlinear electrophoresis as represented by continuous lines (labeled as real). As it can be seen in **Figure S4**, there is significant differences between the estimations, demonstrating the importance of nonlinear electrophoresis on particle velocity and migration time. It is important to also note that **Figure S4** shows the nonlinear behavior of the real electrophoretic velocity with the electric field. Although the effect is slight (this effect is more pronounced in **Figure S6** below), all the velocities represented as continuous lines in **Figure S4** do not follow a linear behavior with the electric field. This isa clear representation of nonlinear electrophoretic velocity effects.

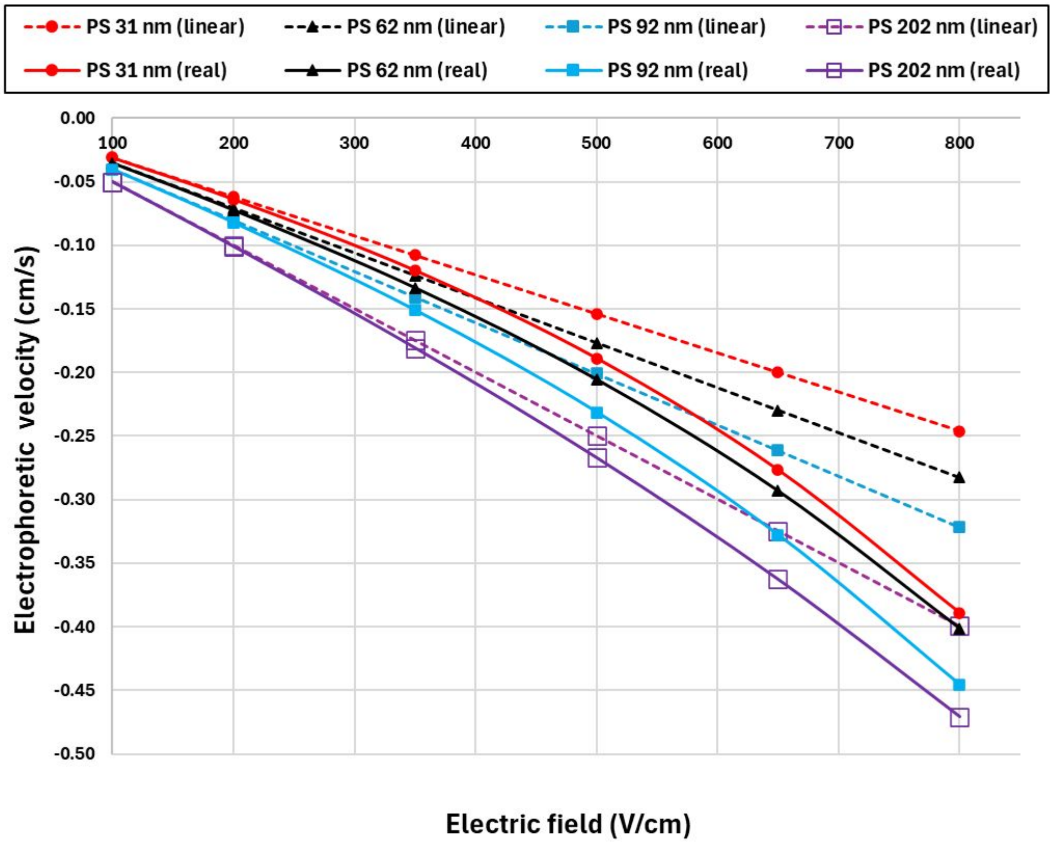

Figure S4. (a) Plot of electrophoretic velocity as a function of the electric field for four types of PS nanoparticles. It was not possible to characterize the nonlinear electrophoretic migration of the PS 303 nm particles. Electrophoretic linear velocity, represented as dotted lines, neglects the effect of nonlinear electrophoresis, while Electrophoretic real velocity, represented as continuous lines considers the effects of nonlinear electrophoresis. To aid visualization, the following markers were employed: filled circle for the PS 31 nm particles, triangle for the PS 62 nm particles, filled square for the PS 92 nm particle and large unfilled square for the PS 202 nm particles.

Figure S5 illustrates the effect of nonlinear electrophoresis for the three PMMA particles and the PP particles studied in this work. The results are more pronounced for the PMMA particles, as the magnitude of their mobility of nonlinear electrophoresis is much higher than that of the PS particles (see **Table 1**). As seen in **Figure S3**, there is a significant difference between the CE linear velocities (represented as dotted lines) estimated neglecting nonlinear electrophoresis ($v_{CE,\,linear} = v_{eof} + v_{e,l}$) and CE real velocities (represented as continuous lines) which also consider the contribution of nonlinear electrophoresis ($v_{CE,\,linear} = v_{eof} + v_{e,l} + v_{e,nl}$). In the case

of three types of PMMA particles, the effect of nonlinear electrophoresis is so strong, that the overall CE real velocity decreases and event turns negative for the PMMA 219 nm particle (data with filled square markers), while the CE linear velocity, keeps increasing as a function of the electric field. Several reports in the literature have illustrated this behavior or particle velocity turning negative as results of nonlinear electrophoresis effects, the results in this work are in full agreement with these published reports.[6,8–10] The stark difference between the "linear" and the "real" CE velocities values showcases the importance of considering nonlinear electrophoresis effects on particle migration in CE systems.

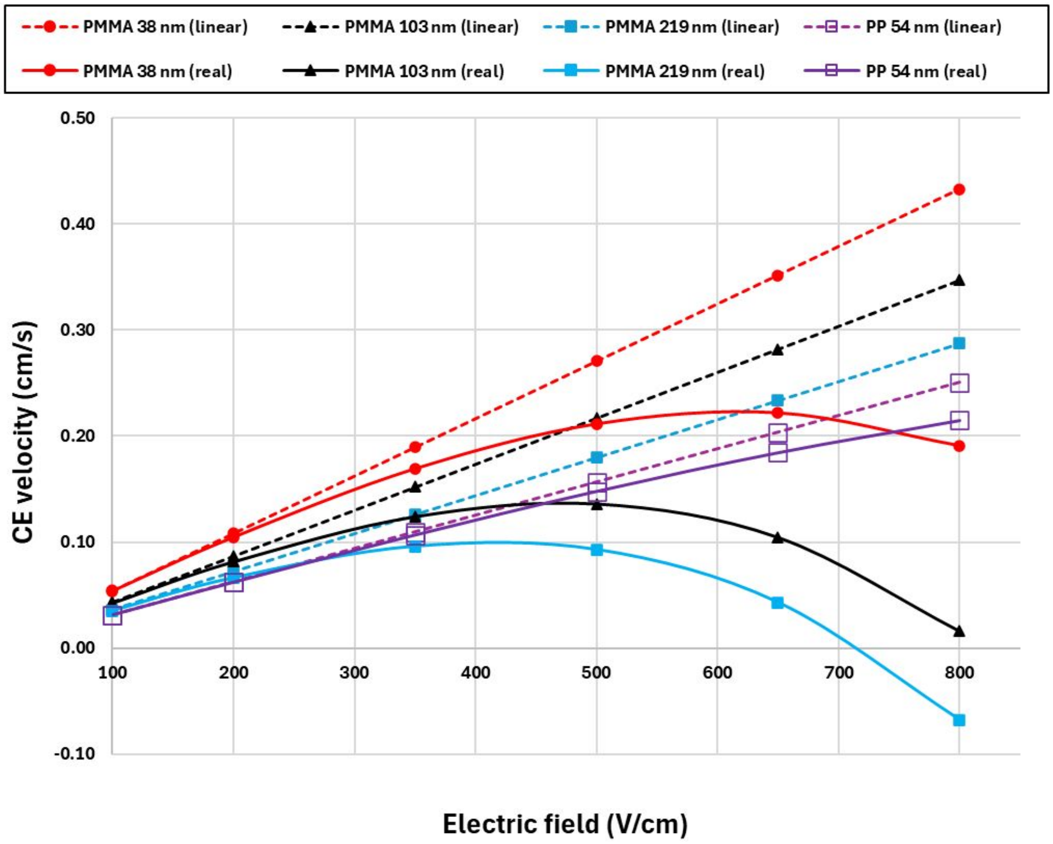

Figure S5. (a) Plot of CE velocity as a function of the electric field for three types of PMMA nanoparticles and the PP 54 nm particle. It was not possible to characterize the nonlinear electrophoretic migration of the PE 56 nm particles. CE linear velocity, represented as dotted lines, neglects the effect of nonlinear electrophoresis, while CE real velocity, represented as continuous lines considers the effects of nonlinear electrophoresis. To aid visualization, the following markers were employed: filled circle for the PMMA 38 nm particles, triangle for the PMMA 103 nm particles, filled square for the PMMA 219 nm particle and large unfilled square for the PP 54 nm particles.

To further highlight the importance of nonlinear electrophoresis, a plot of the electrophoretic velocity as a function of the electric field was also prepared, these results are in **Figure S6**. Similar to **Figure S5**, the plot contains three PMMA particles and the PP particles, where the linear electrophoretic velocity estimations ($v_{e,\,linear} = v_{e,l}$) without considering nonlinear electrophoresis are represented in dotted lines (labeled as linear) and the real electrophoretic velocity estimations ($v_{e,\,real} = v_{e,l} + v_{e,nl}$) considering the effects of nonlinear electrophoresis as represented by continuous lines (labeled as real). The results in **Figure S6**, show a significant difference on velocity magnitude when nonlinear electrophoresis is considered, these results also showcase the nonlinear behavior of the electrophoretic velocity with the electric field as all the real electrophoretic velocity predictions, shown as continuous lines, do not follow a linear behavior. The results illustrate how significant nonlinear electrophoresis effects are at higher electric fields.

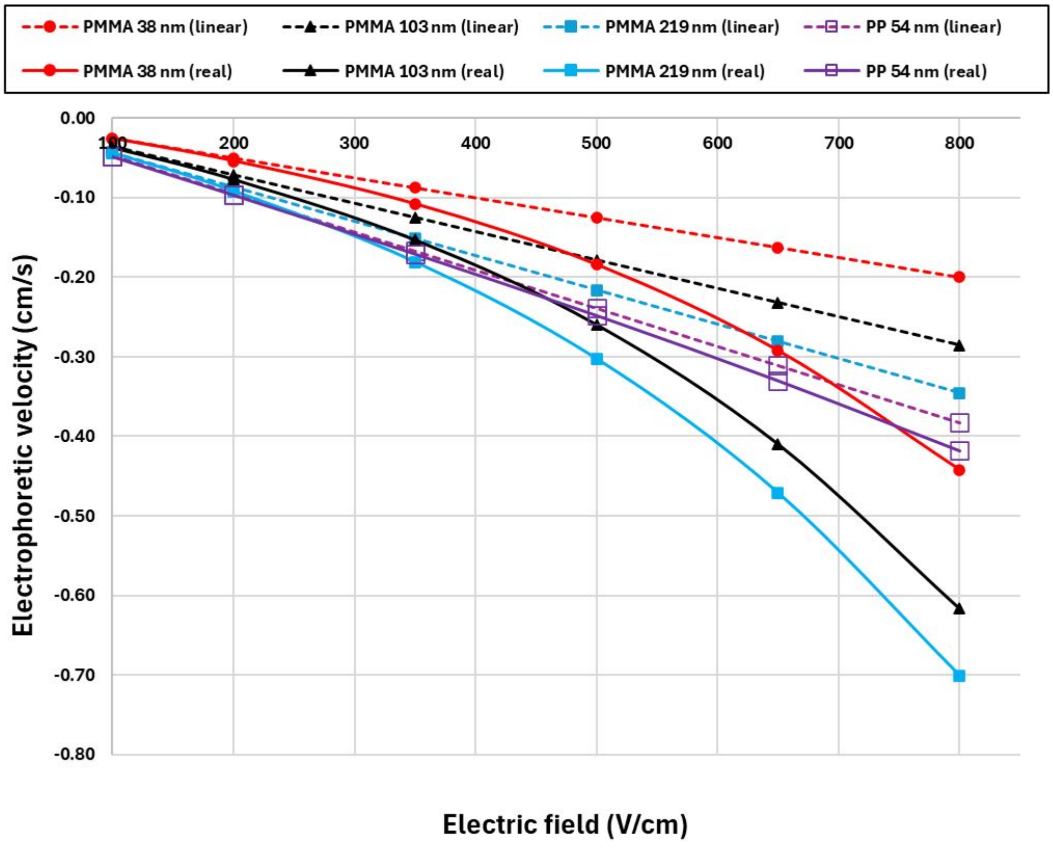

Figure S6. (a) Plot of electrophoretic velocity as a function of the electric field for three types of PMMA nanoparticles and the PP 54 nm particle. It was not possible to characterize the nonlinear electrophoretic migration of the PE 56 nm particles. Electrophoretic linear velocity, represented as dotted lines, neglects the effect of nonlinear electrophoresis, while CE real velocity, represented as continuous lines considers the effects of nonlinear electrophoresis. To aid visualization, the following markers were employed: filled circle for the PMMA 38 nm particles, triangle for the PMMA 103 nm particles, filled square for the PMMA

219 nm particle and large unfilled square for the PP 54 nm particles.

The velocity data for all particles studied here is included in **Tables S4-S5**. **Table S4** contains the data for the PS nanoparticles where CE experiments were carried out under E = 460 V/cm, whilst **Table S5** contains the data for PMMA, PE and PP nanoparticles with CE experiments under E = 560 V/cm. At can be seen from **Tables S4-S5**, the contributions from nonlinear electrophoresis to particle migration are important, when comparing the linear to the nonlinear electrophoretic velocities, the nonlinear contributions can be as much as 59,4% of the linear electrophoretic velocity. These results are in agreement with the plots in **Figures S3-S6**, that compares CE velocities with and without considering nonlinear electrophoresis effects. As illustrates by **Figures S3-S6** and **Tables S4-S5**, nonlinear electrophoresis effects are significant and must be considered for accurate estimations of particle velocity and retention time.

Table S4. Velocity contributions of all three electrokinetic phenomena for the PS nanoparticles, where CE experiments were carried out under an electric field of 460 V/cm.

Particle	Diameter (nm)	v_{CE} (cm/s)	v_{eof} (cm/s)	$v_{e,l}$ (cm/s)	$v_{e,nl}$ (cm/s)	Ratio $v_{e,nl}/v_{e,l}$ (%)
**PS**	31 (1)	0,195	0,364	-0,142	-0,027	19,19
**PS**	62 (1)	0,179	0,364	-0,163	-0,023	13,87
**PS**	92 (1)	0,156	0,364	-0,185	-0,024	12,75
**PS**	202 (1)	0,121	0,364	-0,230	-0,014	5,91
**PS**	303 (1)	0,088	0,364	-0,275	NM*	NM*

(1) Measurement obtained with TEM (details on Table S1)

* Not measurable under current conditions for this particle size.

Table S5. Velocity contributions of all three electrokinetic phenomena for the PMMA, PE and PP nanoparticles, where CE experiments were carried out under an electric field of 560 V/cm.

Particle	Diameter (nm)	v_{CE} (cm/s)	v_{eof} (cm/s)	$v_{e,l}$ (cm/s)	$v_{e,nl}$ (cm/s)	*Ratio* $v_{e,nl}/v_{e,l}$ *(%)*
**PMMA**	38 (1)	0,244	0,467	-0,140	-0,083	59,4
**PMMA**	103 (1)	0,153	0,467	-0,200	-0,114	56,9
**PMMA**	219 (1)	0,103	0,467	-0,242	-0,122	50,5
**PP**	54 (1)	0,187	0,467	-0,268	-0,012	4,60
**PE**	65 (1)	0,280	0,467	-0,277	NM*	NM*

(1) Measurement obtained with DLS

* Not measurable under current conditions for this particle size.

References

(1) Xiong, B.; Pallandre, A.; Le Potier, I.; Audebert, P.; Fattal, E.; Tsapis, N.; Barratt, G.;

Taverna, M. Electrophoretic Mobility Measurement by Laser Doppler Velocimetry and Capillary Electrophoresis of Micrometric Fluorescent Polystyrene Beads. *Anal. Methods* **2012**, *4* (1), 183–189. DOI: 10.1039/c1ay05598d.

(2) Ramírez-García, G.; d'Orlyé, F.; Gutiérrez-Granados, S.; Martínez-Alfaro, M.; Mignet, N.; Richard, C.; Varenne, A. Functionalization and Characterization of Persistent Luminescence Nanoparticles by Dynamic Light Scattering, Laser Doppler and Capillary Electrophoresis. *Colloids Surf., B* **2015**, *136*, 272–281. DOI: 10.1016/j.colsurfb.2015.09.022.

(3) Vanifatova, N.; Spivakov, By.; Kamyshny, A. Comparison of Potential of Capillary Zone Electrophoresis and Malvern's Improved Laser Doppler Velocimetry for Characterisation of Silica Nanomaterials in Aqueous Media. *Int. J. Nanoparticles* **2011**, *4* (4). DOI: 10.1504/IJNP.2011.043499.

(4) Schnitzer, O.; Yariv, E. Nonlinear Electrophoresis at Arbitrary Field Strengths: Small-Dukhin-Number Analysis. *Phys. Fluids* **2014**, *26* (12). DOI: 10.1063/1.4902331.

(5) Dukhin, S. S. Electrokinetic Phenomena of the Second Kind and Their Applications. *Adv. Colloid Interface Sci.* **1991**, *35*, 173–196. DOI: 10.1016/0001-8686(91)80022-C.

(6) Cardenas-Benitez, B.; Jind, B.; Gallo-Villanueva, R. C.; Martinez-Chapa, S. O.; Lapizco-Encinas, B. H.; Perez-Gonzalez, V. H. Direct Current Electrokinetic Particle Trapping in Insulator-Based Microfluidics: Theory and Experiments. *Anal. Chem.* **2020**, *92* (19), 12871–12879. DOI: 10.1021/acs.analchem.0c01303.

(7) Mishchuk, N. A.; Barinova, N. O. Theoretical and Experimental Study of Nonlinear Electrophoresis. *Colloid J.* **2011**, *73* (1), 88–96. DOI: 10.1134/S1061933X11010133.

(8) Antunez-Vela, S.; Perez-Gonzalez, V. H.; De Peña, A. C.; Lentz, C. J.; Lapizco-Encinas, B. H. Simultaneous Determination of Linear and Nonlinear Electrophoretic Mobilities of Cells and Microparticles. *Anal. Chem.* **2020**, *92* (22), 14885–14891. DOI: 10.1021/acs.analchem.0c03525.

(9) Lomeli-Martin, A.; Azad, Z.; Thomas, J. A.; Lapizco-Encinas, B. H. Assessment of the Nonlinear Electrophoretic Migration of Nanoparticles and Bacteriophages. *Micromachines* **2024**, *15* (3), 369. DOI: 10.3390/mi15030369.

(10) Ernst, O. D.; Vaghef-Koodehi, A.; Dillis, C.; Lomeli-Martin, A.; Lapizco-Encinas, B. H. Dependence of Nonlinear Electrophoresis on Particle Size and Electrical Charge. *Anal. Chem.* **2023**, *95* (16), 6595–6602. DOI: 10.1021/acs.analchem.2c05595.
